# Gut Mesenchymal Stromal Cells in Immunity

**DOI:** 10.1155/2017/8482326

**Published:** 2017-02-28

**Authors:** Valeria Messina, Carla Buccione, Giulia Marotta, Giovanna Ziccheddu, Michele Signore, Gianfranco Mattia, Rossella Puglisi, Benedetto Sacchetti, Livia Biancone, Mauro Valtieri

**Affiliations:** ^1^Department of Infectious, Parasitic and Immune-Dependent Diseases, Istituto Superiore di Sanità, Rome, Italy; ^2^Department of Hematology, Oncology and Molecular Medicine, Istituto Superiore di Sanità, Rome, Italy; ^3^Gastroenterology Unit, Tor Vergata University, Rome, Italy; ^4^Sbarro Institute for Cancer Research and Molecular Medicine and Center of Biotechnology, College of Science and Technology, Temple University, Philadelphia, PA, USA

## Abstract

Mesenchymal stromal cells (MSCs), first found in bone marrow (BM), are the structural architects of all organs, participating in most biological functions. MSCs possess tissue-specific signatures that allow their discrimination according to their origin and location. Among their multiple functions, MSCs closely interact with immune cells, orchestrating their activity to maintain overall homeostasis. The phenotype of tissue MSCs residing in the bowel overlaps with myofibroblasts, lining the bottom walls of intestinal crypts (pericryptal) or interspersed within intestinal submucosa (intercryptal). In Crohn's disease, intestinal MSCs are tightly stacked in a chronic inflammatory milieu, which causes their enforced expression of Class II major histocompatibility complex (MHC). The absence of Class II MHC is a hallmark for immune-modulator and tolerogenic properties of normal MSCs and, vice versa, the expression of HLA-DR is peculiar to antigen presenting cells, that is, immune-activator cells. Interferon* gamma* (IFN*γ*) is responsible for induction of Class II MHC expression on intestinal MSCs. The reversal of myofibroblasts/MSCs from an immune-modulator to an activator phenotype in Crohn's disease results in the formation of a fibrotic tube subverting the intestinal structure. Epithelial metaplastic areas in this context can progress to dysplasia and cancer.

## 1. Introduction

### 1.1. Mesenchymal Stromal Cells

Mesenchymal stromal cells (MSCs) represent the second stem cell population residing in bone marrow (BM) [1, 2], wherein they provide both the reservoir for skeletal cell turn-over and “the soil” for parenchymal cells, that is, hematopoietic stem cells (HSCs), “the seeds.” MSCs are endowed with extensive proliferation, three months or longer in vitro; the ability to differentiate into at least four lineages, osteoblasts, chondroblasts, adipoblasts, and reticular stromal cells supporting HSCs; and the capability to produce large amounts of multiple growth factors [1, 2] underlying their antiapoptotic, immune-regulator, and nurturing potential [1–3]. Although MSCs were first discovered in bone marrow, other tissue-specific MSCs have been recently recognized and studied based on their antigen expression signatures and cell differentiation spectra. These tissue-specific MSCs partly overlap with pericytes and display ubiquitous distribution in human tissues as structural cells [3, 4]. We first isolated and characterized MSCs in bone marrow [1]. Subsequently, we isolated and banked tissue-specific MSCs from amnion, foetal liver, tonsil, dental pulp, lung, gut [2, 3], and dermal connective tissue. Bowel MSCs have been classified functionally and by smooth muscle actin expression [2, 3]. Pericryptal myofibroblasts are in close contact with intestinal epithelial stem cells (ISCs) at the bottom of Lieberkühn crypts, wherein they nurture, regulate, and maintain intestinal stem cells (ISCs) similar to BM-MSCs and HSCs in the hematopoietic niche. Pericryptal myofibroblasts hierarchically represent the highest intestinal MSC rank, that is, share the most “stemness” features with BM-MSCs. Conversely, intercryptal MSCs represent a differentiated progeny of pericryptal myofibroblasts and are mainly devoted to structural functions. MSC presence in the intestine might play a pivotal role in modulating the intestinal immune system [5, 6] in terms of tolerance toward commensal microbiota [7, 8] and proficiency in recognition and elimination of the pathogens eventually breaching the mucosal barrier. Due to the symbiotic nature of the human gut, wherein prokaryote microbiota largely outnumber eukaryotic cells, the importance of adequate homeostasis [2] is paramount.

### 1.2. Crohn's Disease

Crohn's disease (CD) is clinically characterized by recurrent and migrating episodes of chronic inflammation of the digestive mucosa, leading to social discomfort and bleeding, evolving from mucosal oedema, thickening, and metaplasia, to granulomas, transmural lesions, and, finally, stenosis and fistulae. Usually CD is surgically treated, but residual areas with metaplasia can progress to dysplasia and, ultimately, to adenocarcinomas. Although the pathogenesis of CD is complex and still, for the most part, obscure [9, 10], deregulation of both adaptive and innate immunity [11, 12] is known to lead, in genetically predisposed hosts, to an inappropriate response to intestinal microbiota. The intestinal epithelium is arranged in villi and crypts to maximize its surface and is composed of ciliated epithelial cells and other specialized cell types such as goblet cells, producing a continuous layer of protective mucus and Paneth cells releasing *α*-defensins. Intercellular and tight junctions among cells lining the intestinal mucosa contribute to sealing the intraluminal microbiota from the underlying lamina propria, while still allowing both the passage of secretory IgA and the stretching of dendritic cell (DC) projections for continuous sampling and monitoring of luminal content. The intestinal lamina propria contains a variety of immune cells either interspersed—mainly innate immune neutrophils, antigen presenting cells (APCs), and natural killer (NK)/T*γ*/*δ* cells—or organized in secondary lymphoid structures like Peyer's patches (adaptive immune B and T cells). Adaptive T cells include proinflammatory T-helper cells (Th1, Th17) and anti-inflammatory T cells (Th2, Treg). The layer of smooth muscle cells underlying the lamina propria, that is, the muscularis mucosa, is discontinued only by blood and lymphatic vessels smallest branches. The mesenteric lymph nodes represent additional secondary lymphoid structures, allowing massive and on-demand lymphocyte expansion. In CD, the structural integrity of the intestinal epithelium is ruptured, allowing excessive amounts of bacteria and oftentimes other pathogens from the CD-altered consortium to engorge immune cells in the underlying lamina propria [13]. As a consequence, Th1 and Th17 cell inflammatory response is triggered, characterized by the release of inflammatory cytokines (mainly TNF-*α*, IL-1*α*, IFN*γ*, IL-23, and IL-17), expansion and recruitment of more proinflammatory cells, enlargement of lamina propria, increased vascularization, and loss of tissue integrity. Gut epithelium itself seems to exert a selective acting role in coordinate T-cell responses toward some segmented filamentous bacteria (SFB) [14]. Microbiota comprises 100 trillion commensal bacteria of more than 1,000 species in the human gut [15], profoundly affecting the immune profile of the host, including the balance between proinflammatory T-helper cells (Th1, Th17) and anti-inflammatory T cells (Th2, Treg). Th1 cells are mainly producing IFN*γ*. An abnormal Th1 response, triggered by increased mucosal levels of IL-18 and IL-12, has been associated with CD while a Th2 response is characterized by secretion of IL-4, IL-5, and IL-13. Th17 cells are able to produce IL-17 and IFN*γ* depending on the context. Treg cells have a similar secretory plasticity and comprise a series of subsets, including CD4+, CD25+, and FoxP3+ cells, and induced-Treg. Comprehension of the mechanisms eventually involved in the regulation, by MSCs, of IFN*γ*-producing Th17 cells or Treg cells, deserves future study. Moreover, T*γ*/*δ* cells have been reported to contribute to chronic inflammation through a rapid and intense production of IFN*γ* [16, 17]. Recent evidence indicates that, in addition to conventional T cells, T*γ*/*δ* cells may represent a major innate source of IL-17 in response to bacterial infection [18]. Moreover, T*γ*/*δ* cells have been shown to exacerbate chronic inflammation by suppressing Treg cell function [19].

### 1.3. Mesenchymal Stromal Cells Immunity

Under physiological conditions, MSCs are known to modulate the function of diverse types of immune cells, both adaptive [20, 21] and innate [16, 17, 22]. However, only recently has their role in an inflammatory or neoplastic microenvironment undergone scrutiny [23]. MSC exhaustion and failure to control CD chronic inflammation result in their IL33-induced expansion/differentiation into fibrotic scar tissue, which has improperly been defined as “creeping fat” [24]. The understanding of intestinal MSC function in orchestrating gut immune responses might lead to the development of MSC targeted-specific drugs. The interest for MSC role in immunity derived many clinical studies. Out of 25 clinical trials with MSCs in Crohn's disease, 5 were completed with bone marrow MSCs and 7 are active or recruiting, 7 were completed with adipose-derived MSCs and 2 are active/recruiting, 1 was completed and 2 are active/recruiting with umbilical cord MSCs, and, finally, 1 was completed with placental MSCs (https://www.clinicaltrials.gov/). Overall these studies have demonstrated the beneficial effects of MSC systemic administration in refractory CD patients, devoid of toxicity and not requiring the ablation of the recipient's immune system [25]. The efficacy of this replacement therapy suggests that endogenous colonic MSCs could be reverted, damaged, or exhausted in the areas affected by CD, and that the infused MSCs are recruited to the inflamed lesions where they home and eventually restore homeostasis. However, time course studies on MSC action are lacking as well as information on their long-term safety. How MSCs behave or react when exposed to the CD chronic inflammatory milieu, including their liaison with Th/Treg or mucosal T*γ*/*δ* cells [16, 17, 26–28], remains unresolved. Exposure of MSCs to inflammatory cytokines has been defined as licensing, but licensed MSC functional comparisons with naïve MSCs remain inconclusive [29–32].

## 2. Results/Discussion

**Figure 1:**
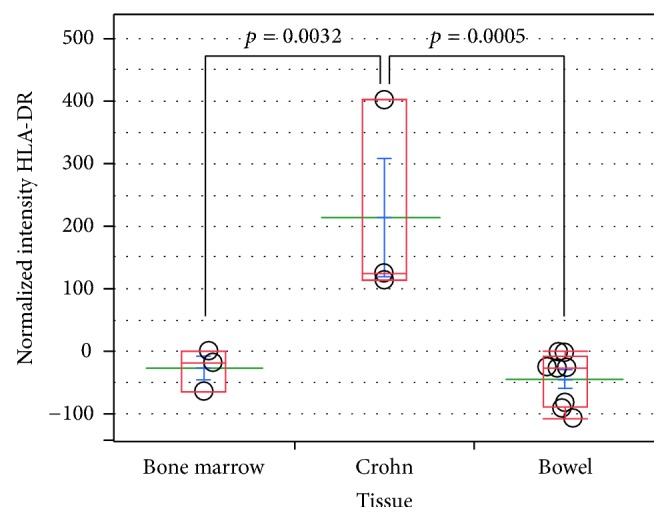
HLA-DR levels in Crohn's-derived versus bowel- and bone marrow-derived MSCs. Dot plot of normalized fluorescence intensities from flow cytometric analysis of HLA-DR expression in Crohn, colon, and bone marrow MSCs. MSCs obtained from colon and bone marrow samples are consistently negative for HLA-DR, a major histocompatibility complex (MHC) Class II antigen, whereas Crohn's-derived MSCs constitutively express statistically significant levels. The antibody used was R-phycoerythrin-labeled mouse anti-human HLA-DR (Becton-Dickinson). Mean fluorescence intensity (MFI) of isotype control was subtracted to MFI from HLA-DR intensity to generate the plotted normalized intensities. *p* values were obtained by performing Student's *t*-test via SAS JMP v10 software (SAS Institute Inc., Cary, NC).

**Figure 2:**
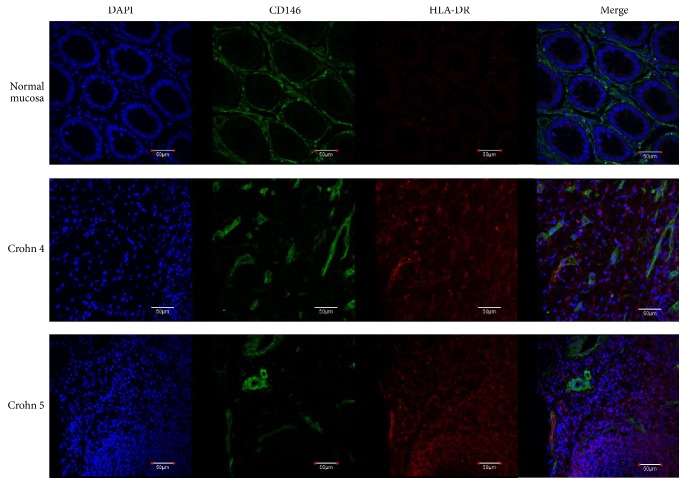
Immunofluorescence analysis of Crohn's disease surgical specimens. In contrast to the typical structure of normal distal intestine mucosa, Crohn's disease sections have a totally subverted architecture wherein the presence of some CD146+ cells expressing high levels of HLA-DR (white arrows) is detected, in close proximity to or interspersed between smaller HLA-DR+ cells. Normal colonic mucosa and Crohn's disease specimens were obtained from patients who signed an informed consent before undergoing surgical resection. Tissues were fixed in PFA 4% o/n at +4°C, embedded, frozen in OCT, and stored at −80°C. Thick sections (15 *μ*m) were cut on a cryotome and processed for immunofluorescence staining. Permeabilization was performed with 0.5% Triton X-100 in PBS for 30′, at room temperature. A blocking step was performed using 40 *μ*g/mL mouse IgG in 3% BSA (PBS) for 2 h at RT, and subsequent incubation with primary antibodies was carried out for 16h at +4°C. Primary antibodies used were FITC conjugated mouse anti-CD146 (Biocytex) and R phycoerythrin-labeled mouse anti-human HLA-DR (Becton-Dickinson). The final step, before mounting the slides, was incubation with a TRITC-conjugated goat anti-PE secondary antibody (Aviva Systems Biology), for 2 h at 37°C. Nuclear staining was executed adding RNAse and DAPI to the secondary antibody incubation mix. Images were acquired with an Olympus FV-1000 spectral confocal microscope, equipped with an UPLFLN 40x 1.30 NA oil immersion objective. Scale bars correspond to 50 *μ*m.

**Figure 3:**
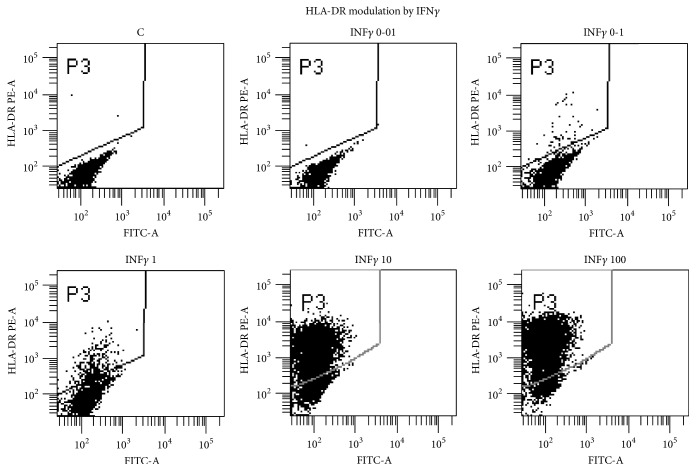
Dot plot analysis of C-MSCs treated with graded amount of IFN*γ* ng/ml for 72 h. Bowel MSCs were stained with PE-labeled HLA-DR mAB (Pharmingen) and analysed with FACSCanto (Becton-Dickinson).

We have recently isolated and banked several MSC lines from most human organs, in both foetal stages and adulthood. Among them 9 bowel MSC lines from normal surgical specimens have been described. They have been extensively characterized and compared to canonical BM-MSC lines, showing a mostly shared identity, yet distinguishable by subtle tissue-specific features [1, 3, 4]. An important hallmark of MSCs, isolated from all tissues, is the absence, in physiological conditions, of HLA-DR expression, which has been linked to both their modulator activity on immune cells and their tolerogenicity as described in [3]. Conversely, our new results show that intestinal MSCs from CD, isolated from endoscopic biopsies, display a constant upmodulation of Class II MHC (Figure S1, in Supplementary Material available online at https://doi.org/10.1155/2017/8482326, BM versus intestinal versus Crohn's MSCs), whose expression might indicate a diversion from the normal MSC immune-modulator function, to an opposite stimulatory APC function. It is noteworthy that HLA-ABC (Class I MHC) expression is unchanged (not shown), being highly expressed by all MSCs (see Table 1 in [3]). HLA-DR overexpression has been observed in 3 intestinal MSC lines (Figure 1, Figures S1 and S2) derived from CD and confirmed in two additional CD bowel sections by confocal microscopy (Figure 2). The CD-MSC expression of Class II MHC and the switch from negative to positive regulation of immune cells [29] might play a significant role in CD pathogenesis. After treatment of normal intestinal MSCs with a series of inflammatory cytokines, we identified IFN*γ* as the main culprit for HLA-DR expression in CD-MSC (Figure 3). Notably, TNF*α* had no effect on Class II HLA expression (data not shown). It is noteworthy that in one patient different biopsies showing increasing levels of inflammation generated MSCs lines with increasing HLA-DR expression (Figure S2, lanes 1 to 4). We can speculate that once the disease has started, and the mucosal barrier integrity has been breached, allowing the bacteria from the intestinal lumen to enter the submucosal layer, immune cells start to fight bacterial cells and elicit an inflammatory response. When this response is prolonged, the high levels of local inflammatory cytokines damage resident MSCs, which, turning on HLA-DR, change their function from immune-modulators to immune-stimulators, as antigen presenting cells, triggering the maintenance of the inflammation in a chronic state. Our interest in intestinal MSCs stems from their identification in foetal bowel [33] and from their potential role in maintaining the intestinal homeostasis [34]. In intestinal sections, resident MSCs form a delicate net enveloping crypts within the lamina propria [3]. Nine subclonal CD146+ MSC lines were derived and characterized from adult intestinal biopsies, in addition to other MSC lines from 6 different human tissues. We identified and characterized two subtypes of gut MSC lines, that is, one subtype lining the bottom walls of intestinal crypts and another forming a cellular network between the crypts as demonstrated in publication [3]. Similar to their BM counterpart, intestinal MSCs are able to modulate immune cells, in vitro, and to inhibit allogeneic T-cell proliferation induced by mature myeloid-derived dendritic cells (MDDCs). This inhibition is mediated by the enhanced production of anti-inflammatory IL-10 and by the inhibition of proinflammatory IL-12 production. Our data highlight a specific role of gut MSCs in controlling intestinal immunity and inflammation [3]. Additionally, in this manuscript, we report the isolation of bowel MSCs from 3 positive staging biopsies for CD and 2 surgical specimens of colon cancer [35]. It would be extremely interesting to study the live interaction of intestinal MSCs with immune cells in space and time under normal and pathophysiological conditions. Such analyses could be performed either in vitro by exploiting human-derived three-dimensional gut tissue models or in vivo by means of mouse models of inflammatory bowel disease.

It is noteworthy that Crohn's disease possible progression to colon adenocarcinoma confers upon it the features of a natural model to study the development of cancer from a chronic inflammation. This includes the role of immune cells operating in a specific microenvironment such as the colonic walls. Colon MSCs maintain their spurious HLA-DR expression in the context of adenocarcinoma as shown in a representative colon cancer MSC line (Figure S2 lane 5). It is apparent that HLA-DR upregulation in gut MSCs represents a link between a chronic inflammatory disease, Crohn's disease, and cancer, colon adenocarcinoma. Emerging evidences on Crohn's disease treated with new molecularly tailored drugs indicate the occurrence of Hepatosplenic T-Cell Lymphoma (HSTCL), which probably follow a different pathogenesis going beyond the purpose of this article.

## Supplementary Material

In Figure S1 we compared, by Flow Cytometry, HLA-DR expression in primary MSCs from bone marrow, as canonical reference MSCs, in normal bowel MSCs and in bowel MSCs with Crohn's Disease (CD). Note the lack of expression in BM-MSCs and bowel MSCs, and the significant expression in CD-MSCs. In Figure S2 we analyzed, by Flow Cytometry, the incresing levels of HLA-DR expression in Crohn's Disease bowel MSCs obtained from progressively increasing levels of inflammaton biopsies from a patient (1-4) and from a colon carcinoma (CC) (5). Note the progressive increase of the indicated percentages of HLA-DR expressing MSCs in CD (1-4) and the high expression in CC (5).
