# Supplementary material for: Gut Mesenchymal Stromal Cells in Immunity
Source: Stem Cells Int. 2017 Feb 28;2017:8482326. doi: 10.1155/2017/8482326 (PMC5350335; doi:10.1155/2017/8482326)
Supplement: Supplementary file 1 — In Figure S1 we compared, by Flow Cytometry, HLA-DR expression in primary MSCs from bone marrow, as canonical reference MSCs, in normal bowel MSCs and in bowel MSCs with Crohn's Disease (CD). Note the lack of expression in BM-MSCs and bowel MSCs, and the significant expression in CD-MSCs. In Figure S2 we analyzed, by Flow Cytometry, the incresing levels of HLA-DR expression in Crohn's Disease bowel MSCs obtained from progressively increasing levels of inflammaton biopsies from a patient (1-4) and from a colon carcinoma (CC) (5). Note the progressive increase of the indicated percentages of HLA-DR expressing MSCs in CD (1-4) and the high expression in CC (5). [file 8482326.f1.pdf]

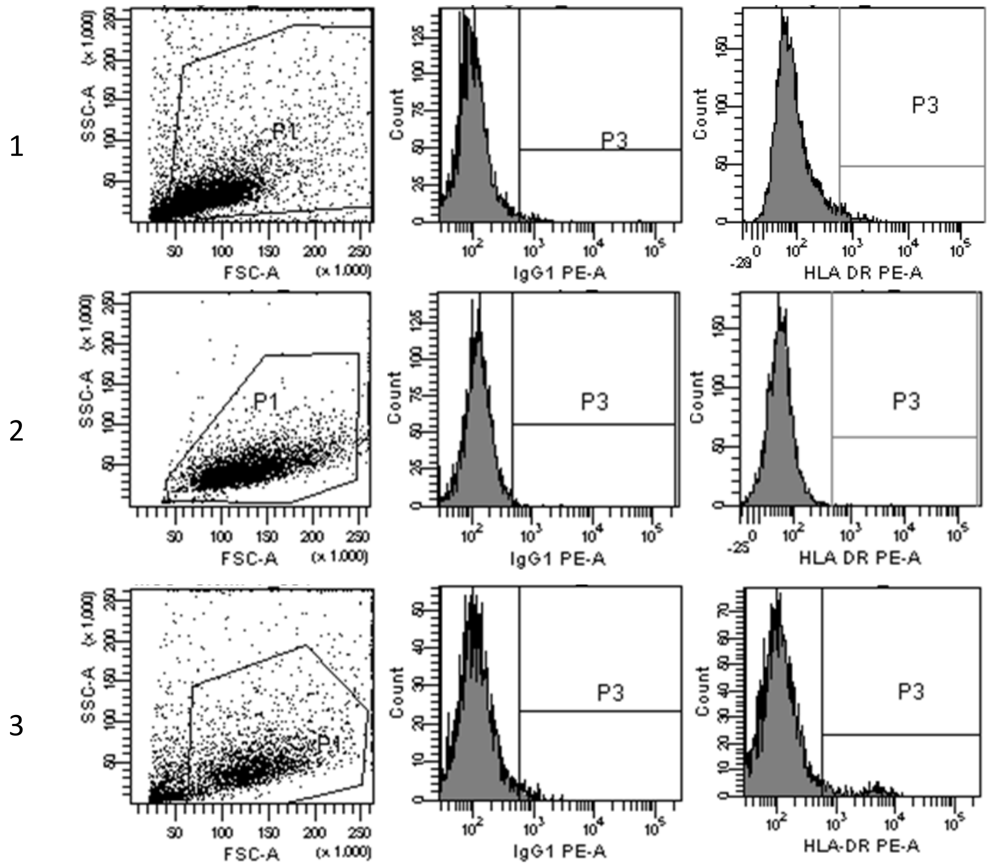

Figure S1. Flow cytometry analysis of HLA-DR expression in MSCs. Data show representative samples of bone marrow (1), bowel (2) and Crohn (3) MSCs, labeled with R-phycoerythrin mouse anti-human HLA- DR (Becton-Dickinson). R-phycoerythrin-labeled mouse IgG1 (R&D System) was used as isotype control.

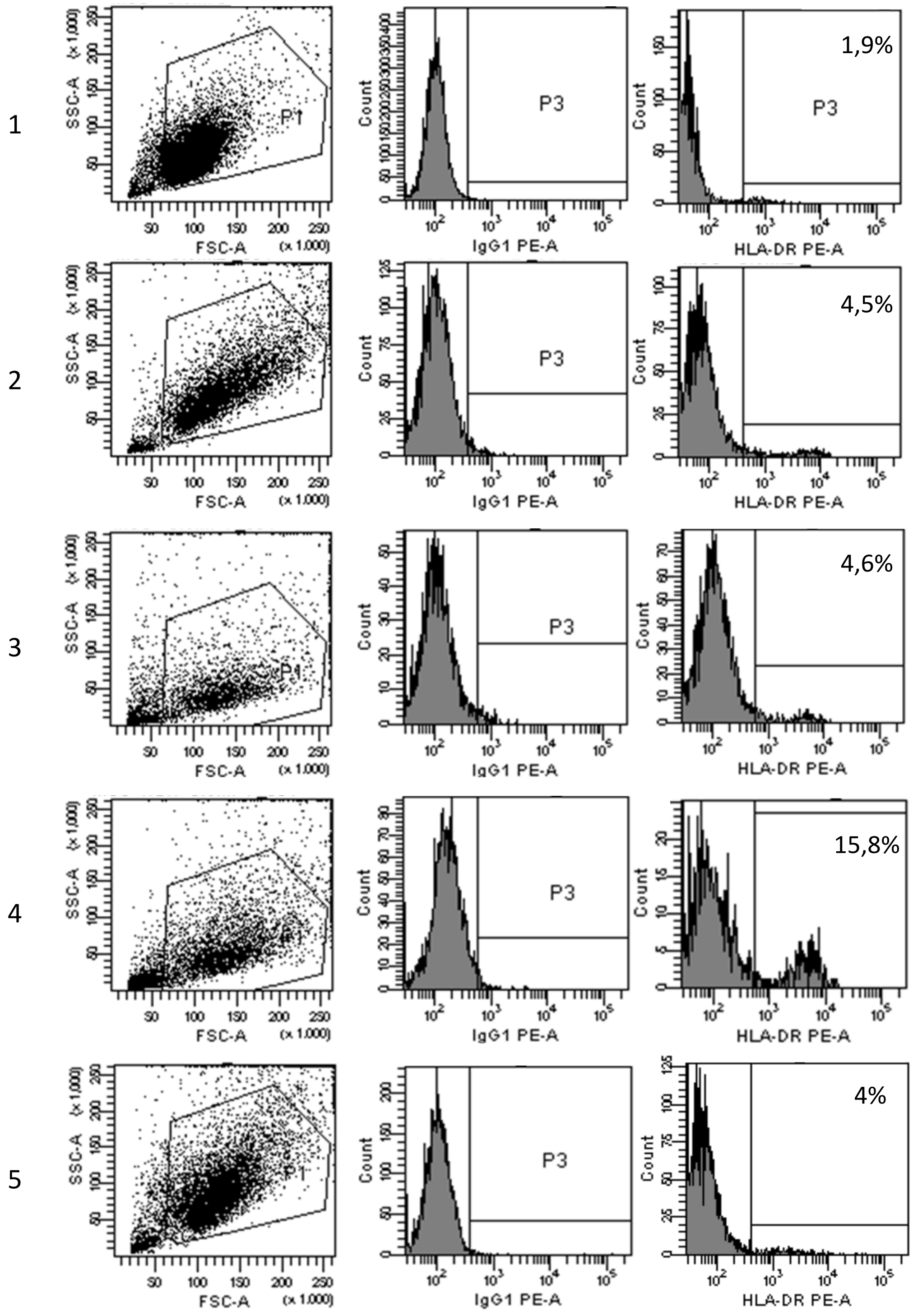

Figure S2. Flow cytometry analysis of HLA-DR expression on intestinal MSCs at increasing levels of inflammation (1-4) and on colon adenocarcinoma (5). Data show the percent values of HLA-DR $^{+}$ cells for each sample. MSCs were labeled with R-phycoerythrin mouse anti-human HLA- DR (Becton-Dickinson),R-phycoerythrin-labeled mouse IgG1 (R&D System) was used as isotype control.
